# Exploring the stability of long intergenic non-coding RNA in K562 cells by comparative studies of RNA-Seq datasets

**DOI:** 10.1186/1745-6150-9-15

**Published:** 2014-07-05

**Authors:** Lei Wang, Dequan Zhou, Jing Tu, Yan Wang, Zuhong Lu

**Affiliations:** 1 State Key Laboratory of Bioelectronics, School of Biological Science and Medical Engineering, Southeast University, Sipailou #2, Nanjing, Jiangsu Province 210096, China; 2 School of Life Science, Lanzhou University, Lanzhou 730000, China; 3 Chengdu Institute of Biology, Chinese Academy of Sciences, Chengdu 610041, China

**Keywords:** RNA Sequencing (RNA-Seq), Long intergenic non-coding RNAs (lincRNAs), K562, RNA stability

## Abstract

**Background:**

The stability of long intergenic non-coding RNAs (lincRNAs) that possess tissue/cell-specific expression, might be closely related to their physiological functions. However, the mechanism associated with stability of lincRNA remains elusive. In this study, we try to study the stability of lincRNA in K562 cells, an important model cell, through comparing two K562 transcriptomes which are obtained from ENCODE Consortium and our sequenced RNA-Seq dataset (PH) respectively.

**Results:**

By lincRNAs analysis pipeline, 1804 high-confidence lincRNAs involving 1564 annotated lincRNAs and 240 putative novel lincRNAs were identified in PH, and 1587 high-confidence lincRNAs including 1429 annotated lincRNAs and 158 putative novel lincRNAs in ENCODE. There are 1009 unique lincRNAs in PH, 792 unique lincRNAs were in ENCODE, and 795 overlapping lincRNAs in both datasets. The analysis of differences in minimum free energy distribution and lincRNA half-life showed that a large proportion of overlapping lincRNAs were more stable than the unique lincRNAs. Most lincRNAs were more unstable than protein-coding RNAs through comparing their minimum free energy.

**Conclusions:**

Identification of overlapping and unique lincRNAs can be helpful to classify the stability of lincRNAs. Our results suggest that overlapping lincRNAs (relatively stable linRNAs) and unique lincRNAs (relatively unstable lincRNAs) might be involved in different cellular processes.

**Reviewers:**

This article has been reviewed by Prof. Oliviero Carugo, Dr. Alistair Forrest and Prof. Manju Bansal.

## Open peer review

Reviewed by Prof. Oliviero Carugo, Dr. Alistair Forrest and Prof. Manju Bansal. For the full reviews, please go to the Reviewers' comments section

## Background

The mammalian genome is extensively transcribed, giving rise to many thousands of non-coding transcripts including both short transcripts (<200 nucleotides in length) and long non-coding RNAs (lncRNAs) (>200 nucleotides in length) [1]. LncRNAs recently have caused more attention because they interact with other biological molecules to regulate diverse cellular processes [2]. According to the location and context in genome, lncRNAs can be classified into intergenic lncRNAs, intronic lncRNAs, sense lncRNAs and antisense lncRNAs [3]. Present studies are mainly focused on intergenic lncRNAs (lincRNAs) located and transcribed from intergenic genomic regions, not only because lincRNAs are more convenient for experimental manipulation and computational analysis without the interference of annotated protein-coding regions than other lncRNAs [4], but also because lincRNAs participate in many cellular processes from embryonic stem cell pluripotency to cell proliferation and cancer progression [5,6].

With large-scale transcriptome sequencing, a growing number of lincRNAs have been identified in mammals [4]. However, only a few of lincRNAs have been functionally characterized. The lincRNA HOTAIRM1 modulates the expression of several HOXA genes, which encode key transcription factors for differentiation of myeloblasts [7]. LincRNA-p21 plays a functional role in p53 response pathways through affecting the expression of numerous genes associating with p53, and recruits ribonucleoprotein K (hnRNP-K) to trigger the regulation of p53-mediated apoptosis [8]. LincRNA-EPS at the nuclear localization is up-regulated in murine erythroid terminal differentiation, which promotes survival of murine erythroblasts [9]. Overall, many lincRNAs can regulate gene expression through specific interactions with other cellular factors, DNA, and other RNA molecules, such as acting in *cis* on neighboring genes or acting in *trans* regardless of gene location [10,11]. Another proposal is that lncRNA genes (including lincRNA) act as enhancer regions and may be incidental by-products of transcription [12].

Previous studies have indicated that RNA stability influences the abundance of transcript and shapes the kinetics of gene induction in intricate gene networks in mammalian cells [13-16]. Therefore, lincRNA stability inevitably affects its function in post-transcriptional regulatory pathways. However, there have been only a few reports about lincRNAs stabilities [7-9,17,18]. Recently, Tani et al. determined the half-lives of transcribed RNAs in whole-genome of HeLa Tet-off (TO) cells by BRIC-Seq and found hundreds of short-lived noncoding transcripts (t1/2 < 4h) [19,20]. Clark et al. found that only a minority of lncRNAs were unstable by confirming the half-lives (half-life < 2h) of about 800 lncRNAs in the mouse Neuro-2a cell line with a custom non-coding RNA array [21]. These studies concluded that mRNAs and lncRNAs (including lincRNAs) have similar half-life distributions [11]. Furthermore, lincRNAs are transcriptionally activated similar to mRNAs, nearly always 5’-capped and 3’-polyadenylated, and are frequently spliced [3,11]. It has been known that short-lived mRNAs were enriched among genes with regulatory functions, whereas long-lived mRNAs with metabolism and structure [22,23]. Like that, lincRNA stability should be closely associated with its physiological function. Although different inhibitor approaches for identifying half-life have been documented in recent years, the stability of lincRNA is poorly understood. It limited functional research of lincRNAs in the complex post-transcriptional regulation.

The human leukemia K562 cells have the potentials for differentiating into erythroid, granulocytic, monocytic and megakaryocytic lineages [24]. It is an important model cell in studying the pluripotency and differentiation of hematopoiesis and leukemogenesis [25,26]. Hence, we investigated the stability of lincRNAs in K562 cells and comparatively analyzed lincRNAs in ENCODE and our dataset named as PH which was sequenced on an Illumina HiSeq™ 2000 with pair-end libraries by RNA-Seq technology. The goals were to (1) improve the lincRNAs analysis workflow to acquire stringent lincRNA set; (2) compare and analyze the lincRNAs in ENCODE and PH datasets; (3) compare the stabilities of coding-protein RNAs and lincRNAs; (4) display lincRNAs distribution in human genome. The results suggest that lincRNAs with different stability may have diverse functions in various biological processes.

## Results

### Pipeline for lincRNA analysis

In order to attain high-confidence lincRNA catalog, there are two main different improvements comparing to previous lincRNA predicting pipeline. (1) The more rigorous set of intergenic transcripts was acquired from the intersection of intergenic transcripts by comparing with four database annotations (Ensembl [27], UCSC [28], Gencode [29] and Refseq [30]), respectively. (2) Putative novel lincRNAs were more strictly attained by filtering coding potential RNAs of the results including iseeRNA [31], CPAT [32], CPC [33] and PhyloCSF [34] (see Methods). Through the lincRNA predicting pipeline, unreliable transcripts were removed and reliable lincRNAs were remained in downstream analysis.

### The features of lincRNAs in PH and ENCODE

**Figure 1:**
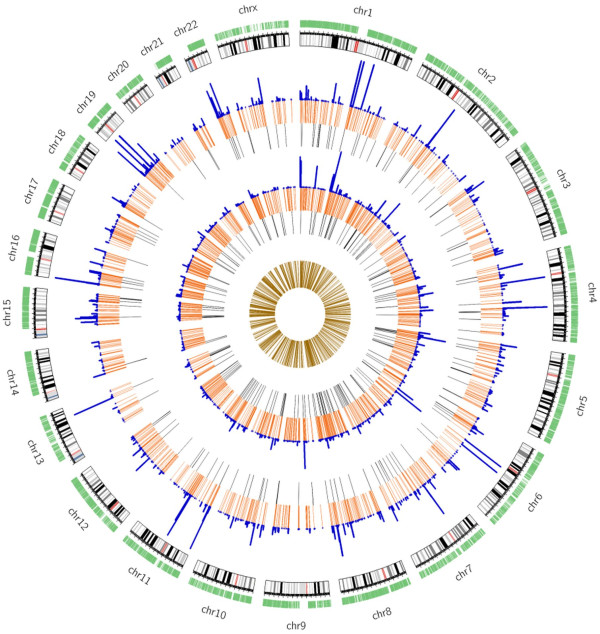
**Distribution of the lincRNAs of K562 cells in human genome.** The outside green circle is lncRNA location of Gencode v18 annotation. The three circles in close proximity to genome are features of the ENCODE lincRNAs (putative novel lincRNAs, black; annotated lincRNAs, orange; the FPKM value (FPKM < 50) for histogram). The inner three circles are features of PH lincRNAs (putative novel lincRNAs, black; annotated lincRNAs, orange; the FPKM value (FPKM < 50) for histogram). The centric circle is the distribution of the overlapping lincRNAs of ENCODE and PH (khaki).

**Figure 2:**
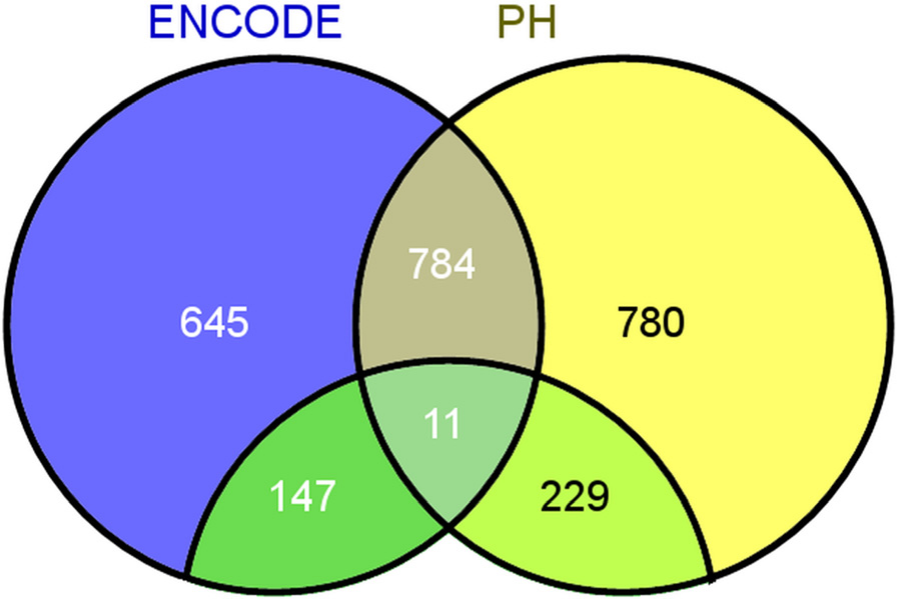
**Venn diagram of lincRNAs between ENCODE and PH.** 645 unique annotated lincRNAs and 147 unique putative novel lincRNAs of ENCODE display at the left; 780 unique annotated lincRNAs and 229 unique putative novel lincRNAs of PH appear at the right; 784 overlapping annotated lincRNAs and 11 overlapping putative novel lincRNAs of both PH and ENCODE display at the middle.

In PH, 1804 lincRNAs were identified, 1564 of which (86.7%) were annotated with the Ensembl or Gencode, and the remaining 240 (13.3%) were putative novel lincRNAs. There were 312 lincRNAs (17.3%) with FPKM (Fragments Per Kilobase of exon model per Million mapped fragments) ≥ 1, including 159 annotated lincRNAs and 153 putative novel lincRNAs. Simultaneously, 1587 lincRNAs were identified in ENCODE, 1429 of which (90.0%) were annotated with the Ensembl or Gencode, and the remaining 158 (10.0%) were putative novel lincRNAs. There were 514 lincRNAs (32.4%) with FPKM ≥ 1, including 455 annotated lincRNAs and 59 putative novel lincRNAs. LincRNAs in K562 cells, whether they were annotated or not in both datasets, were uniformly and prevalently distributed at every chromosome in human genome, even if most of the transcripts were low-abundant (FPKM <1) (Figure 1). By comparing lincRNAs of both datasets, 1009 lincRNAs were unique in PH, 792 lincRNAs were unique in ENCODE (unique lincRNAs present in only PH or ENCODE dataset), and 795 lincRNAs were overlapping in both datasets (overlapping lincRNAs present in both ENCODE and PH datasets) (Figure 2, Additional file 1: Table S1, Additional file 2: Table S2, Additional file 3: Table S3, Additional file 4: Table S4).

### Comparison of minimum free energy and lincRNAs half-lives between PH and ENCODE

**Figure 3:**
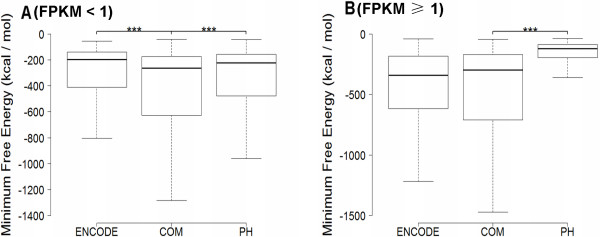
**Comparison of the minimum free energy between the unique and overlapping lincRNAs in both datasets.** Box-and-whisker plot. (Whiskers) 1st–99th percentile, without individual lincRNAs outside this. (Box) 25th–75th percentile. Difference calculated using a nonparametric Mann-Whitney t-test [(***) P < 0.000001]. **(A)** Comparison of the minimum free energy of the unique and overlapping lincRNAs with FPKM < 1. **(B)** Comparison of the minimum free energy of the unique and overlapping lincRNAs with FPKM ≥ 1. (PH, the unique lincRNAs in PH; ENCODE, the unique lincRNAs in ENCODE; COM, the overlapping lincRNAs of both datasets).

**Figure 4:**
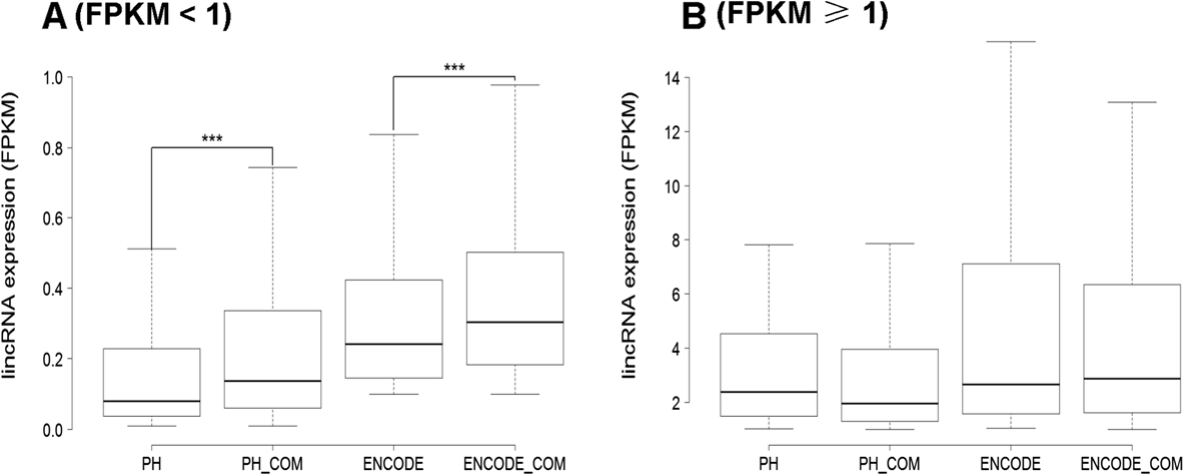
**Comparison of expression level between the unique and overlapping lincRNAs in both datasets.** Box-and-whisker plot. (Whiskers) 1st–99th percentile, without individual lincRNAs outside this. (Box) 25th–75th percentile. Difference calculated using a nonparametric Mann-Whitney t-test [(***) P < 0.000001]. **(A)** Comparison of expressed abundance of the unique and overlapping lincRNAs with FPKM < 1. **(B)** Comparison of expressed abundance of the unique and overlapping lincRNAs with FPKM ≥ 1. (PH, the unique lincRNAs in PH; ENCODE, the unique lincRNAs in ENCODE; PH_COM, the overlapping lincRNAs in PH; ENCODE_COM, the overlapping lincRNAs in ENCODE).

**Figure 5:**
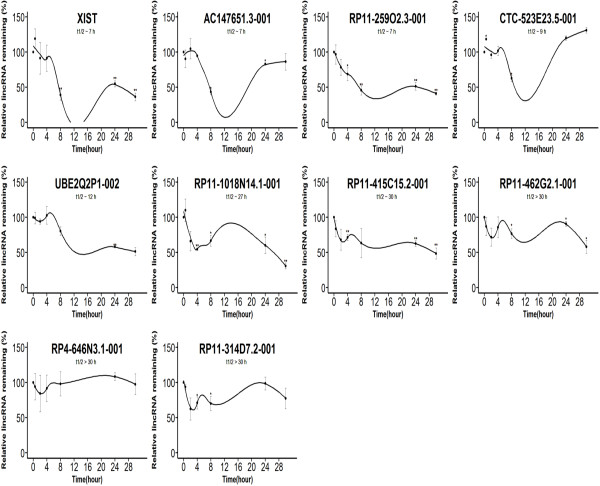
**Stabilities of overlapping lincRNAs in both datasets.** LincRNA decay curves after blocking transcription in K562 with actinomycin D and measuring relative levels of lincRNA remaining relative to a control gene (GAPDH) by qPCR. The relative quantitative values at time 0 h were arbitrarily adjusted to 100%. The fitted curve was modeled using locally weighted polynomial regression (LOESS method). Results are from three biological replicates, and Error bars show standard deviation [(**) P < 0.01; (*) P <0.05, Student’s t-test].

**Figure 6:**
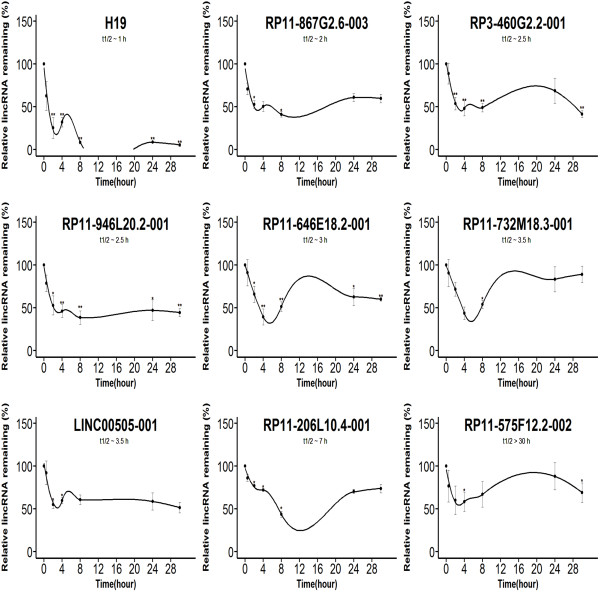
**Stabilities of unique lincRNAs in PH or ENCODE.** LincRNA decay curves after blocking transcription in K562 with actinomycin D and measuring relative levels of lincRNA remaining relative to a control gene (GAPDH) by qPCR. The relative quantitative values at time 0 h were arbitrarily adjusted to 100%. The fitted curve was modeled using locally weighted polynomial regression (LOESS method). Results are from three biological replicates, and Error bars show standard deviation [(**) P < 0.01; (*) P <0.05, Student’s t-test].

Whether lincRNAs expressed with FPKM ≥ 1 or FPKM < 1, the minimum free energy of most overlapping lincRNAs were higher than unique lincRNAs (Figure 3). Although the expressed abundances of the majority of overlapping lincRNAs were higher than unique lincRNAs in the cases of FPKM < 1 (Figure 4), the minimum free energy and expression level (FPKM) are not correlated (Pearson's correlation coefficient < 0.2 in both datasets). Comparing the determined ncRNAs half-lives in Hela cells [19], 12 overlapping lincRNAs were found with half-life > 4 in both datasets, 2 unique lincRNAs in PH and 3 unique lincRNAs in ENCODE. In this study, random 10 overlapping lincRNAs half-lives (including FPKM ≥ 1 and FPKM < 1, Figure 5) and random 9 unique lincRNAs half-lives (including FPKM ≥ 1 and FPKM < 1 from PH or ENCODE, Figure 6) were determined by qPCR after ActD treatment [21]. The results proved that 10 overlapping lincRNAs half-lives are more than 4 hours (3 of which more than 30 hours), while 7 unique lincRNAs half-lives are less than 4 hours.

### Comparison of the minimum free energy of the common protein-coding RNAs and lincRNAs

**Figure 7:**
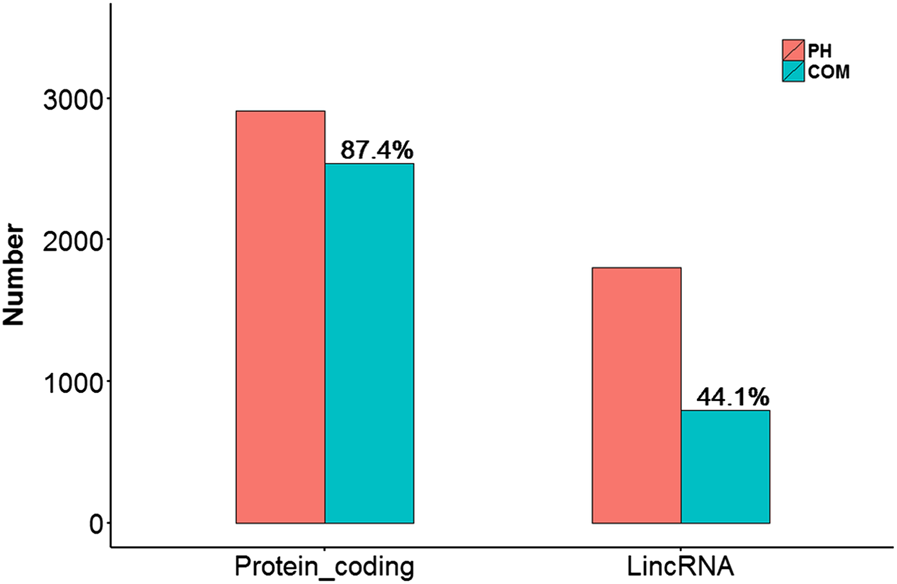
**The number of the protein-coding RNAs and lincRNAs in PH.** 2914 protein-coding RNAs exist in PH, 2546 (87.4%) of which present in both datasets. 1804 lincRNAs exist in PH, 795 (44.1%) appear in both datasets.

**Figure 8:**
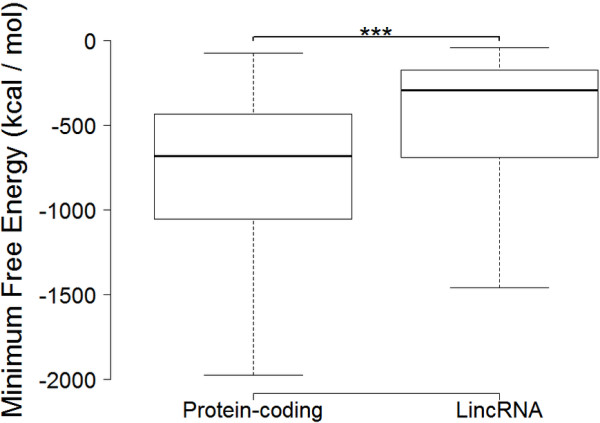
**Comparison of the minimum free energy between the common protein-coding RNAs and lincRNAs.** Box-and-whisker plot. (Whiskers) 1st–99th percentile, without individual transcripts outside this. (Box) 25th–75th percentile. Difference calculated using a nonparametric Mann-Whitney t-test [(***) P < 0.000001].

2914 protein-coding RNAs for the intersection of Cuffcompare’s results with four public database annotations (Ensembl, UCSC, GENCODE and Refseq) were identified in PH, 2546 (87.4%) of which presented in ENCODE. Likewise, 795 lincRNAs (44.1%) in PH appeared in both datasets (Figure 7). By comparing the minimum free energy of the common protein-coding RNAs and lincRNAs of the both datasets, the result showed that the minimum free energy of a large proportion of protein-coding RNAs was significantly higher than lincRNAs (Figure 8).

## Discussion

In this study, the lincRNA analysis workflow was improved to acquire stringent lincRNA set. The method to acquire the intersection of intergenic transcripts is simpler and more convenient than integrating several database annotations by their scripts [4,35]. Moreover, possible novel lincRNAs with uncertain coding potential (TUCPs) which possibly possess protein-coding or small peptides potential, were filtered by four softwares (iseeRNA, CPAT, CPC and PhyloCSF), respectively. Thereby more reliable putative novel lincRNAs were attained.

The genome encodes far more lincRNAs than previously known, and pervasively transcriptional lincRNAs might play widespread roles in gene regulation and other cellular processes [36]. In the study, thousands of lincRNAs including annotated lincRNAs and putative novel lincRNAs were identified in both datasets through our lincRNA predicting pipeline. And, those lincRNAs were transcribed from thousands of locations at every chromosome in human genome.

The sequence of nucleotides of an RNA molecule carries the functional information. As more lincRNAs are identified, it will become important to study lincRNA sequences and their secondary structures to reveal mechanisms of lincRNA functions through establishing structure-function relations [11,37-40]. These roles including sensory, guiding, scaffolding and allosteric capacities derive from folded modular domains in lincRNAs [41]. Minimum free energy secondary structure, which is predicted with minimizing the free energy of a conformation according to a thermodynamic mode, is functionally important [42]. The lower the value of minimum free energy is, the more energy unfolding RNA requires, so that RNA can be unfolded to be bound by some factors (including miRNA) to trigger degradation processes. Hence, minimum free energy can partly reflect lincRNA’s stability. Previous studies showed that protein-coding RNAs are more stable than lncRNAs (including lincRNAs) [43], which agree with our result that the secondary structures of a large proportion of protein-coding RNAs are more stable than lincRNAs by the analysis of minimum free energy.

The large variation in lncRNA stability is associated with their functional diversity [4,25,44]. Furthermore, lncRNAs can cluster into the same decay profiles which may be regulated by the same post-transcriptional regulatory pathways and/or contain similar regulatory sequences [21]. Previous studies propose that lncRNAs with more structural elements are more stable [21]. Through the analysis of Gene Ontology (GO) terms with differences in mRNA half-life distribution, Tani et al. found that short-lived mRNAs (t_1/2_ < 4 h) were significantly enriched in implicated regulatory functions, and long-lived mRNAs (t_1/2_ ≥ 4 h) were disproportionately represented implicated housekeeping functions [19]. Furthermore, some lincRNAs with short half-lives (t_1/2_ < 4 h) were well-known regulatory lincRNAs, and several ncRNAs with long half-lives (t_1/2_ ≥ 4 h) were involved in housekeeping functions [19], as is the case with mRNAs. The median lncRNA (including lincRNA) half-life is 3.5 h (mean 4.8 h) [21], so unstable lincRNA is determined with half-life < 4 h, and stable lincRNA is determined with half-life ≥ 4 h.

Although the functions of a large proportion of both overlapping and unique lincRNAs in PH and ENCODE datasets were mysterious, we suggested their functions by correlating the stability of lincRNA to the functional categories. In this study, most overlapping lincRNAs were more stable than unique lincRNAs regardless of FPKM by the analysis of differences in minimum free energy distribution and lincRNA half-lives. Moreover, there was no correlation between lncRNA (including lincRNA) expression and half-life [21], similar to the result that lincRNA expression and minimum free energy were not correlated. Highly stable mRNAs often encode highly stable proteins with “housekeeping” functions [45]. Similarly, relatively stable lincRNAs with long half-lives may serve “housekeeping” roles. Stable lincRNAs may avoid degradation for undertaking some functions through various mechanisms, such as the stable secondary structure and interactions with RNA-binding proteins. The overlapping lincRNA XIST (half-life ~ 7 h) mediates X-chromosome inactivation in *cis* by recruiting a chromatin-modifying complex to specific sites [46]. It is a potent suppressor of hematologic cancer in mice, which may account for that human malignancies sometimes show X chromosome aneuploidies [17]. Additionally, MALAT-1, intergenic ~7 kb single exon transcript, has a highly conserved tRNA-like sequence at the 3' end and process to generated a short tRNA-like ncRNA mascRNA [47]. It is stable in human B cells and Hela (half-life > 7 h) and has been found to regulate alternative splicing of endogenous target genes in the light of the information of lncrnadb [48].

On the other hand, relatively unstable lincRNAs with short half-lives may be expressed in narrow time windows in response to external stimuli, which is vital for reflecting regulatory functions [19,21]. Low stabilities are characterized in many transcription factor mRNAs, and transcription factors can regulate gene expression in response to environmental signals through activating or repressing target genes [23,49]. Unstable lincRNAs such as H19 [18], HOTAIR [50] and GAS5 [51], could act almost immediately after transcription without producing a functional gene product in the nucleus, so they might not require too long half-lives. Furthermore, unstable RNAs (including lincRNAs) associating with chromatin binding proteins suggested unstable lincRNA would be suitable for regulating gene expression [10,21]. The unique lincRNA H19 (half-life ~ 1 h) is abundantly expressed during embryonic development and down-regulated after birth and can regulate processing of miR-675 [18,52]. Additionally, HOTAIR with a half-life < 4 h in human Hela cells [19], has active role in modulating the cancer epigenome by binding to the polycomb repressive complex 2 (PRC2) [50]. GAS5 with a half-life < 4 h in human Hela cells [19], is a ribo-repressor by binding DNA domain of the glucocorticoid receptor to influence cell survival and metabolic activities during starvation [51].

## Conclusions

We improved lincRNAs predicting pipeline to attain high-confidence lincRNAs and showed that lincRNAs are ubiquitous transcribed in K562 cells. By comparing both RNA-Seq datasets of K562 cells to explore the stability of lincRNAs, a large proportion of overlapping lincRNAs were more stable than unique lincRNAs by the analysis of differences in minimum free energy distribution and lincRNA half-lives. These results implied that overlapping lincRNAs (relatively stable linRNAs) and unique lincRNAs (relatively unstable lincRNAs) could be associated with different functions, which will facilitate future experimental and computational investigations about lincRNA for leukemia disease.

## Methods

### Cell culture

K562 cell line was cultured in RPMI 1640 medium (GIBCO, Life Technologies) supplemented with 10% (v/v) fetal bovine serum, 100 units/ml Penicillin, and 100 μg/ml Streptomycin (P/S) (GIBCO, Life Technologies) in incubator (5% CO_2_, at 37°C).

### RNA extraction, illumina library construction and sequencing

Total RNA from K562 cells was prepared using Trizol reagent (Invitrogen.USA). Subsequently, they were used for mRNA purification and library construction with the Truseq™ RNA Sample Preparation Kit v2 (Illumina, San Diego, CA, USA) following the manufacturer’s instructions. Our sample was named PH that was sequenced on an Illumina HiSeq™ 2000 (Illumina) with pair-end libraries in Encode Genomics Bio-Technology Co. (Suzhou, China).

### Preprocessing RNA-Seq datasets

Transcriptome reconstruction of PH and ENCODE datasets of K562 cells by RNA-Seq was performed respectively using rigorous read set through a sliding window filtering the average quality values within the window less than 20 and the length of reads less than 35 bp by Trimmomatic [53]. After quality control, we obtained 90.7 million 2*100-base paired-end reads generated by Illumina Hiseq2000 sequencing on polyadenylated selected (Poly-A^+^) RNAs. On the other hand, ENCODE RNA-Seq dataset was incorporated 112.3 million 2*76-base paired-end reads generated by Illumina GAIIx sequencing on Poly-A^+^ RNAs from NCBI Gene Expression Omnibus (GEO) database with accession number GSM765405.

### Obtaining annotated and putative novel lincRNAs

**Figure 9:**
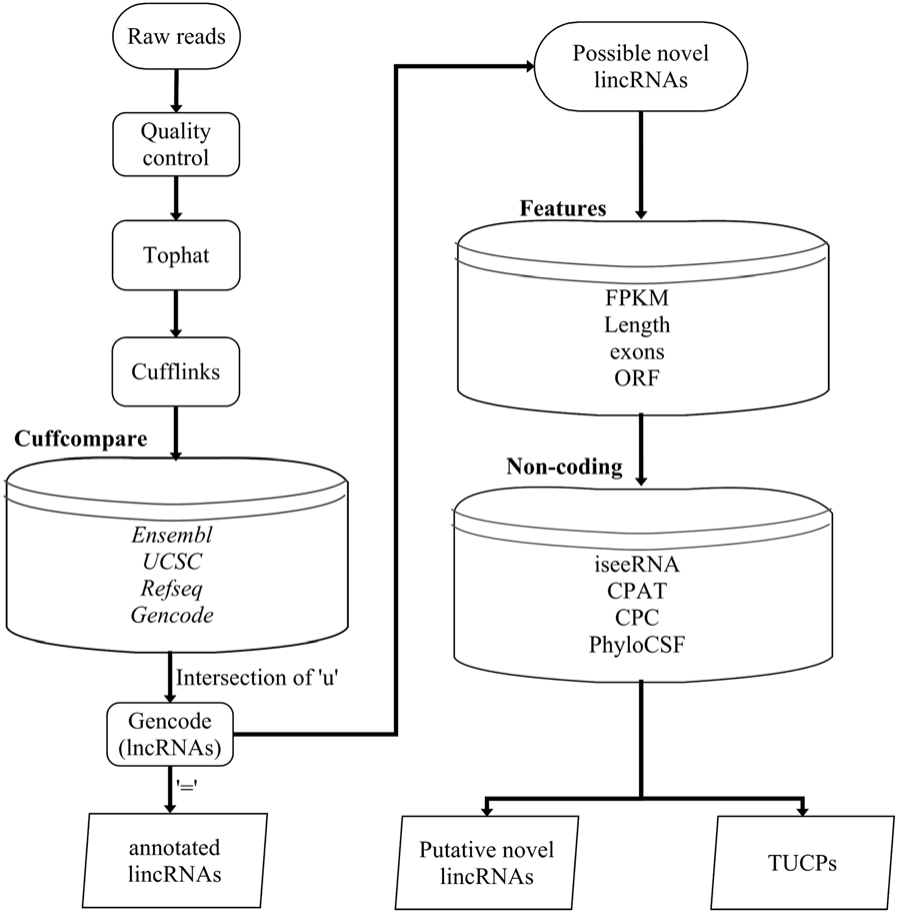
**An overview of our pipeline for obtaining annotated lincRNAs and putative novel lincRNAs from RNA-Seq dataset.** TUCPs, the transcripts of uncertain coding potential; ‘u’, intergenic transcript in Cufflinks; ‘=’, complete match of intron chain in Cufflinks (see Methods).

Quality-control reads were aligned by TopHat (v2.0.7) [54], and transcripts of PH and ENCODE datasets were reconstructed by Cufflinks (v2.0.2) [55] with the Ensembl annotation, respectively. Because of strand-specific of ENCODE by RSeQC script [56], fr-firststrand library type was performed for ENCODE by Cufflinks. To eliminate all annotated non-lincRNA transcripts, the intersection of transcripts of the ‘u’ category (unknown, intergenic transcript in Cufflinks) was attained using cuffcompare script with four public databases annotations (annotated protein-coding genes, microRNAs, rRNAs, tRNAs and pseudogenes), including Ensembl (Homo_sapiens.GRCh37.70.gtf), UCSC (hg19), Gencode (gencode.v15.annotation.gtf.gz) and Refseq (ref_GRCh37.p10_top_level.gff3) respectively. That is, the intersection of intergenic transcripts was acquired apart from all annotated non-lincRNA annotations of four databases. Annotated lincRNAs were acquired through the intersection of intergenic transcripts to run Cuffcompare script again with Gencode lncRNAs annotation (gencode.v18.long_noncoding_RNAs.gtf.gz). The remaining transcripts were possible novel lincRNAs. Then, possible novel lincRNAs were filtered on the basis of some characteristics including FPKM value (Due to the low expression of lincRNAs, we considered to acquire more lincRNAs based on the density distribution of lincRNAs. ENCODE, FPKM ≥ 0.1; PH, FPKM ≥ 0.01), length ( ≥ 200 nt), ORF (< 100 codons) and exonic number ( ≥ 2). After that, putative novel lincRNAs were acquired based on non-coding potential by integrating the results of four softwares including iseeRNA (noncoding), CPAT (no), CPC (noncoding) and PhyloCSF (score < 100) (Figure 9).

iseeRNA, a lightweight SVM-based program, is designed for computational identification of lincRNAs from high-throughput transcriptome sequencing data. CPAT, which overcomes several intrinsic pairwise and multiple alignments limitations, uses logistic regression model based on ORF size, ORF coverage, Fickett TESTCODE and Hexamer bias. CPC relys on pairwise alignment to assess the protein-coding potential of a transcript based on six biologically meaningful sequence features. PhyloCSF uses a multi-species nucleotide sequence alignment to calculate the phylogenetic conservation score, which is likely to represent a protein-coding region.

### Minimum free energy

Minimum free energy secondary structure is assessed by minimum free energy (thermodynamic free energy). RNAfold predicts minimum free energy and minimum energy secondary structures using a loop-based energy model and dynamic programming algorithm [57,58].

### Half-life verification using quantitative real-time PCR (qRT-PCR)

For experiment of half-life, cells were grown to ~50% confluency before RNA polymerase activity was blocked by 10μg ml^−1^ actinomycin D (sigma) in DMSO. Transcription inhibition of K562 was carried out for 30 hours, and cells were harvested at time 0 h, 0.5 h, 2 h, 4 h, 8 h, 24 h and 30 h, respectively.

Total RNA samples at different time were prepared using Trizol reagent (Invitrogen, Carlsbad, CA, USA). One microgram of RNA was reverse transcribed into cDNA using Reverse Transcriptase M-MLV (TAKARA, Japan), and diluted by 1:5 for RT-PCR assay. The assay was performed by using SYBR Premix Ex Taq (TAKARA, Japan) on ABI 7500 Real-Time PCR System. GAPDH was used as an internal control. 2^-ΔΔCt^ values were calculated for each gene to show the fold change. All qRT-PCR reactions were performed in three biological replicates.

We determined the lincRNA half-life by calculating the time when the fold change of expression abundance reached half of the initial expression abundance (0 h time point). Decay profile was modeled using locally weighted polynomial regression (LOESS method) (http://www.r-project.org).

## Reviewers’ comments

We appreciate the reviewer’s comments from Prof Oliviero Carugo, Dr Alistair Forrest and Prof Manju Bansal. We have revised the manuscript accordingly.

### Reviewer #1 (First Round): Prof Oliviero Carugo, University of Vienna, Austria

#### Major comments

The main assumption of this manuscript is that overlapping lincRNAs (those that are found both in the ENCODE dataset and in the PH dataset) have functions different from unique lincRNAs (those that are found only in one dataset). In fact, on the basis of the data shown in the manuscript, this is an epistemological axiom (if not a postulate) that might be questioned. The experimental observation that five (5) unique lincRNAs are less stable than seven (7) overlapping lincRNAs is in fact insufficient to conclude that there is a functional difference.

Authors’ response: *We have revised the whole paper to demonstrate our ideas. We showed that a large proportion of overlapping lincRNAs were more stable than the unique lincRNAs through the analysis of differences in minimum free energy distribution. Furthermore, the experiments of lincRNA half-lives also supply the result. Through not only the half-lives of overlapping and unique lincRNAs but also the well-studied lincRNA with known half-life and previous studies about RNA stablility, we suggested that overlapping lincRNAs (relatively stable linRNAs) and unique lincRNAs (relatively unstable lincRNAs) could be associated with different functions.*

As a consequence, the entire manuscript is highly speculative and this must be clearly pointed out in the discussion.

Authors’ response: *We have added some evidences to supply our ideas and moderated some speculations which were insufficient evidences in the discussion.*

In the chapter entitled “Obtaining annotated and putative novel lincRNAs”, in the “Methods” section, a more detailed description must be provided to allow one to reproduce the algorithm. If this is too long, a supplementary file may be submitted (with some examples).

Authors’ response: *We have re-written the lincRNA analysis workflow in the “Methods” section.*

Also the introductory section should be modified. The first part of the “Background” section is a good introduction about the stability of non-coding RNAs and its functional implications. Some additional emphasis should be put on the relationships with cancer and diseases. Otherwise, the mention to medicalissues is inappropriate. The last part of the “Background” section, which is a short summary of the experiments and of the results of the manuscript, should be slightly expanded to be more easily understood by the reader. In its present form, it is too short and it becomes, inevitably cryptic.

Authors’ response: *Thanks for the comments of reviewer. We have re-written the introduction and revised the whole article.*I cannot inspect Figure 8 (resolution is too small).

Authors’ response: *We have adjusted it in the revised version.*

### Minor comments

Section “Background” line 43 – I am expecting that the Authors describe briefly which cancers and which diseases are related to lncRNAs. The first sentence in this section is otherwise too vague for a scientific publication.

Authors’ response: *We have revised the introduction and deleted the first sentence.*

Section “Background” line 46 – Please, check if the expression “… that are proved the importance …” is correct. Perhaps it should be “… that are proven to be important …”

Authors’ response: *We have revised the introduction and deleted the sentence.*

Section “Background” line 62 – Please, check if the expression “… Clark et al found only a minority …” is correct. Perhaps it should be “…Clark et al found that only a minority …”.

Authors’ response: *Thanks, we have corrected it in the revised version.*

Section “Background” line 68 – Please, check if the expression “… we improve lincRNA …” is correct. Perhaps it should be “… we improve the lincRNA …”

Authors’ response: *We have corrected it in the revised version.*

Section “Background” lines 68-71 – These two sentences, which are a sort of summary of the experiments described in this manuscript, are a bit confusing. First, the Authors write that they compared “different RNA-Seq datasets. Then, they write that they compared “both RNA-Seq datasets”. The question is: are they “several” or just “two”? Moreover, the Authors should describe briefly which are these datasets, how they were assembled, validated, compared, etc. Just few sentences should be enough to improve the readability of the manuscript.

Authors’ response: *We revised introduction and described the improved lincRNAs workflow in the “Methods” section.*

Section “Background” lines 72-73 – The expression “through randomly testifying … unique lincRNAs” is not clear. The Authors should re-write it.

Authors’ response: *The sentence has been re-written according to the reviewer’s suggestion*

Section “Background” line 74 – The expression “coinciding” might be “in agreement with”.

Authors’ response: *We have deleted the sentence in the revised manuscript.*

Section “Background” line 70 – The Authors should justify why they selected K562 cells.

Authors’ response: *It has been revised according to the reviewer’s suggestion.*

Section “Background” lines 76-77 – The sentence “Therefore, we suggest … lincRNA stability” is unclear and should be re-written.

Authors’ response: *We rewrote it in the revised manuscript.*

Section “Results and discussion” line 85 – A reference to Cufflinks is mandatory. And it is also necessary to mention what is that (briefly).

Authors’ response: *We have added the cited paper in the revised manuscript.*

Section “Results and discussion” line 86 – Probably it would be better to write simply “by considering the annotations present in XXX” or “by using the software XXX” and “(see Methods for details)”. In its present form and without references, this list of resources is not really readable.

Authors’ response: *We added the cited paper in the revised manuscript.*

Section “Results and discussion” lines 90-91 – The Authors should justify why their method is more effective than alternative methods based on the integration of several databases. In fact, I understand that it is simpler. But I am not sure it is more effective.

Authors’ response: *We have revised it.*

Section “Results and discussion” lines 103-104 – The Authors mention “four public database annotations”. They should also indicate their names and where they can be found.

Authors’ response: *It has been revised according to the reviewer’s suggestion.*

Section “Results and discussion” line 104 – The Authors mention “PH” here for the first time: it is necessary to define it explicitly. If I am not wrong, it is defined only in the Abstract and this is not sufficient.

Authors’ response: *PH was defined in the “Methods” section.*

Section “Results and discussion” lines 109-113 – The Authors describes the“minimum free energy”. This should be described better and it is necessary to cite the computational procedure used to compute this thermodynamic quantity.

Authors’ response: *We have revised it in the “Methods” section according to the reviewer’s suggestion.*

Section “Results and discussion” lines 116-120 – These sentences repeat the“Background” section and should be removed (or moved to the introductory section).

Authors’ response: *These sentences been deleted according to the reviewer’s suggestion in the revised manuscript.*

Section “Results and discussion” lines 126-127 – The expression “Comparing the both datasets, …” might be inappropriate and might be even removed.

Authors’ response: *It has been revised according to the reviewer’s suggestion.*

Section “Results and discussion” line 128 – RNAfold can be used to compute something and it cannot be calculated.

Authors’ response: *We have deleted the sentence in the revised manuscript.*

Section “Results and discussion” line 132 – The verb “testified” might be inappropriate. Why not “determined experimentally”?

Authors’ response: *It has been revised according to the reviewer’s suggestion.*

Section “Results and discussion” line 134 – The “5 unique lincRNA” were taken from PH or from ENCODE?

Authors’ response: *It has been revised according to the reviewer’s suggestion.*

Section “Results and discussion” line 136 – A reference to “qPCR after ActD treatment” is necessary.

Authors’ response: *We have added the cited paper in the revised manuscript.*

Section “Results and discussion” lines 137-138 – A expression “… which is coordinated …” might be inappropriate. Perhaps it might be changed into “… in agreement with …”.

Authors’ response: *We have deleted the sentence in the revised manuscript.*

Section “Results and discussion” line 143 – The verb “express” might be “may be expressed”. Section “Results and discussion” line 151 – The verb “is” might be inserted between “It” and “highly”.

Authors’ response: *It has been revised according to the reviewer’s suggestion.*

Section “Results and discussion” lines 150-157 – Caution: this discussion is highly speculative and it should be re-written. It is necessary to clearly indicate that these are mere suppositions of the Authors that are not based on any new results.

Authors’ response: *We have re-written in the revised manuscript.*

Section “Results and discussion” line 164 – The expression “FPKM” should be indicated extensively.

Authors’ response: *We have re-written the sentence in the revised manuscript.*

Section “Methods” line 188 – The verb “were” might be “was”.

Authors’ response: *Thanks, we corrected it in the revised version.*

Section “Methods” line 196 – The sentence “Total RNA was extracted as described above” might be deleted.

Authors’ response: *We have deleted the sentence in the revised manuscript.*

Section “Methods” line 231 – “We” might be “we”.

Authors’ response: *We are sorry about this mistake, and have corrected it in the revised version.*

Reference number 8. The journal name should be abbreviated. The same goes for several other references.

Authors’ response: *We fully checked the manuscript and revised similar question.*

Reference number 34. Volume and pages are missing. The same happens also in some other reference.

Authors’ response: *We fully checked the manuscript and revised similar question.*In the right part of Figure 1, the box named “Non-coding” might be modified by listing the four programs in the same order used to describe them in lines 93-100 of the section “Results and discussion”: iseeRNA, CPC, PhyloCSF, CPAT. In the present figure, CPAT is the second program and not the fourth.

Authors’ response: *We have modified the description about Figure*9*in the “Methods” section.*The caption of Figure 2 is unclear. It should be re-written.

Authors’ response: *We have revised it.*Figure 3 – Which units are used the measure the free energy? The data shown in the figure are taken from PH or Encode? It is also necessary to write what are the thick horizontal black lines and what the error bars indicate. In fact, although most of the readers will understand this figure, it is mandatory to write an exhaustive legend.

Authors’ response: *It has been revised according to the reviewer’s suggestion.*In Figure 4, “Encode” should be “ENCODE”.

Authors’ response: *We are sorry about this mistake, and have corrected it in the revised version.*Figure 5 – See the observations about Figure 3.

Authors’ response: *It has been revised according to the reviewer’s suggestion.*Figures 6 and 7 – These curves have very different shapes. Might the Authors try to describe them and write something about the differences?

Authors’ response: *It has been revised according to the reviewer’s suggestion.*

### Reviewer #1 (Second Round): Prof Oliviero Carugo, University of Vienna, Austria

The revised version of the manuscript is considerably better than the original version. However, in my opinion, there is still a very modest direct and experimental evidence of the difference between overlapping and unique lincRNAs. As a consequence, the conclusions are extremely speculative.

Authors’ response: *To explore the stability of two separated classes of lincRNAs by comparing both RNA-Seq datasets of K562 cells, we have analyzed minimum free energy distribution and measured the half-lives of the selected lincRNAs. The results supported the conclusion that the overlapping lincRNAs show more stable than the unique lincRNAs in K562 cell. Actually, the conclusion could be accepted in a general consideration that the overlapping lincRNAs expressed in both datasets should have more probability to be detected than the unique lincRNAs only expressed in one single dataset. Therefore, the lincRNAs existing in two datasets were likely more stable than those existing in one single dataset.*

### Reviewer #2 (First Round): Dr Alistair Forrest, Omics Science Center, Japan

#### Major comments

1. "The method is simpler and more effective than integrating several database annotations by their own scripts [29,30]." Unless you provide benchmarking this is an unsupported statement. Should be removed.

Authors’ response: *We did not compare the effectiveness without their scripts, however, our pipeline is simpler and more convenient than integrating several database annotations by the scripts.*

2. "our sequenced RNA-Seq dataset (PH)." Why is the dataset called PH?

Authors’ response: *We are really grateful to the reviewer’s carefulness. A series of RNA-Seq datasets were sequenced in K562 cells by PMA or hemin treatment. PH was untreated RNA-Seq dataset.*

3. The main point of the paper seems to be that reproducible lncRNAs have higher free energy and inferred secondary structure than lncRNAs only observed in one dataset. I think this is likely to just reflect abundance. More highly expressed lncRNAs are more likley to be observed in multiple datasets. Weakly expressed lncRNAs are less likely. The authors could perhaps strengthen their story by looking at the relationship between expression level and stability by breaking the lncRNAs into several bins (low, mid, high) and examine the free energy (and half-life) in box plots.

Authors’ response: *Thanks for the reviewer’s helpful suggestions. We have taken this advice and analyzed the relation of the minimum free energy and expression level in the revised manuscript. However, they were not correlated, similar to no correlation between lncRNA (including lincRNA) expression and half-life.*

4. "highly expressed in ENCODE, but hardly expressed in PH." For these kind of statements it is very important to explain what RNA-seq protocol was used for ENCODE and PH. If the methods do not match this may explain why you see differences.

Authors’ response: *We appreciate the reviewer’s suggestion. ENCODE and PH datasets were sequenced following the manufacturer’s instructions of Illumina. And we analyzed them using the same method (seeMethods).*

5. "Unstable lincRNAs are very sensitive, respond rapidly when transcription changes and act almost immediately after transcription without producing a functional gene product in the nucleus." Unsupported statement. You do not demonstrate that unstable lncRNAs are inherently 'rapid responders', neither do you demonstrate 'without producing a functional gene product'. The manuscript should be re-read critically examining whether your statements are supported by your analysis or from a primary reference.

Authors’ response: *We added the cited paper and revised the sentence.*

6. "Pervasive transcription of lincRNAs in K562 cells". This section doesn't really add anything. "514 lincRNAs (FPKM > =1) in ENCODE (80 for FPKM > =10), whilst there were 312 lincRNAs (FPKM > =1) in PH(30 for FPKM > =10). 89 overlapping lincRNAs of both datasets expressed with FPKM > =1." This does in no way suggest pervasive.

Authors’ response: *We really thank for the reviewer’s question. We have re-written the section. The distribution and expression of lincRNA were showed in the revised Figure*1*.*

### Minor comments

1. "LncRNAs, located and transcribed from intergenic genomic regions that are proved the importance in cancer by genome-wide studies, are named intergenic lncRNAs (lincRNAs)," Do you mean the lncRNAs or the genomic regions where they are found are associated with cancer? The references 5, 6 do not directly correspond to a cancer link.

Authors’ response: *We have rewrote the introduction and deleted the sentence.*

2. "lincRNA" and "lncRNA" are used interchangably in the paper. lincRNA corresponds to 'intergenic', whereas lncRNA are just long. You should make a definition and stick to one, probably lncRNA.

Authors’ response: *LincRNA is a subgroup of lncRNA. The features of lncRNA also are showed for lincRNA. Some cited papers were associated with lncRNA, however, lncRNA included lincRNA.*

3. "It is found that a large proportion of overlapping lincRNAs are more stable than unique lincRNAs". I suggest the authors not use the term 'overlapping lincRNAs' as this suggests genomic overlap. I think what the authors mean is 'lncRNAs observed in multiple K562 datasets are more stable than lncRNAs unique to one K562 dataset'

Authors’ response: *We have annotated overlapping lincRNAs and unique lincRNAs in the “Results” section. We compared PH and ENCODE datasets to attain overlapping and unique lincRNAs in venn diagram (Figure*2*). Unique linRNAs presented in only PH or ENCODE dataset, and overlapping lincRNAs presented in both ENCODE and PH datasets.*

4. "In light of this, we acquired a great deal of intergenic transcripts involved possible novel lincRNAs and annotated lincRNAs with Ensembl or Gencode". Unclear what "transcripts involved possible novel" means.

Authors’ response: *It has been revised according to the reviewer’s suggestion.*

5. "Hexamer usage bais" Bias

Authors’ response: *Thanks, we have corrected it in the revised version.*

6. "1804 lincRNAs were indentified" Identified

Authors’ response: *We are sorry about this mistake, and have corrected it in the revised version.*

7. "We randomly testified the half-lives of 7" tested

Authors’ response: *We have revised it.*

### Reviewer #2 (Second Round): Dr Alistair Forrest, Omics Science Center, Japan

#### Major points

1. Ok. The authors have removed the sentence, still the section is entitled 132

"Improved pipeline for lincRNA analysis". I would change to just "Pipeline for lincRNA analysis" as still you have provided no evidence that the pipeline is 'improved'.

Authors’ response: *It has been revised according to the reviewer’s suggestion.*

2. FIX. The authors must add a sentence at the very first use of PH explaining that PH is (PMA or Hemin treated K562s).

Authors’ response: *We have added the annotation at the very first use of PH in the revised manuscript. In this study, PH was only named for our RNA-Seq dataset of untreated K562 cells.*3. FIX. Figure 4 should not be split into A and B on 1 FPKM. Only one set of boxplots should be shown.

Authors’ response: *Figure*4*splits into A and B on 1 FPKM in order to correspond to the minimum free energy (A and B in Figure*3*). It can clearly illuminate the cases of FPKM ≥ 1 and FPKM < 1.*

4. OK. Actually GSM765405 was sequenced using the CSHL RNA-seq protocol not Truseq. However should be comparable.

Authors’ response: *In this study, we have compared the protein-coding RNAs from both datasets by different sequencing library construction methods. We found that 2546 (87.4%) protein-coding RNAs in PH also presented in ENCODE, which showed that the results from CSHL RNA-seq protocol and Truseq are comparable.*

5. OK

6. OK

#### New minor comments

1. line 139 PhloCSF [34]. PhyloCSF

Authors’ response: *We have corrected the spelling mistake in the revised version*

2. expressive abundance (FPKM). expression level or transcript abundance

Authors’ response: *It has been revised according to the reviewer’s suggestion.*

### Reviewer #3 (First Round): Prof Manju Bansal, Indian Institute of Science, India

The authors evaluate the stability of long intergenic non-coding RNA (lincRNA) in human K562 cell using the RNA-seq data. Two datasets of lincRNA are used in the study. The authors developed a pipeline to enriche lincRNA compared to that seen in ENCODE dataset. The stability of the lincRNA in these two dataset is compared, using mfe predicted by secondary structure program RNAfold. The reason to carry out this comparison is not clearly explained. It is no surprise that non-coding regions have lower stability than protein-coding regions.

Authors’ response: *The secondary structure of lincRNA is important for its stability. We estimated the stabilities of overlapping and unique lincRNAs by bioinformatic method (minimum free energy). Furthermore, through the analysis of minimum free energy, our result agreed with previous report that lincRNA have lower stability than protein-coding RNA. That is, it was verification that the stability of lincRNA could be assessed using minimum free energy.*

Further, the dataset is divided into overlapping and unique. Stability study shows that overlapping lincRNAs are more stable than unique lincRNAs. The explanation for the observed difference is not clearly stated. I cannot understand why lincRNA identified in two datasets (overlapping) should be more stable.

Authors’ response: *We showed that a large proportion of overlapping lincRNAs were more stable than the unique lincRNAs through the analysis of differences in minimum free energy distribution and lincRNA half-lives.*

RNA half-life studies are carried out on few lincRNAs under each category. The findings of the RNA half-life studies, using very few samples (5-7), cannot be generalized for > 700 odd lincRNAs in each group. The conclusion that lincRNA stability may be related to function is already shown in Ref [26,28]. Overall, the conclusions made by the authors are rather weak or already estabilshed.

Authors’ response: *Since lots of experiments may need too much time, we have added some experiments of lincRNA half-lives. We have revised the conclusions and suggested that overlapping lincRNAs (relatively stable linRNAs) and unique lincRNAs (relatively unstable lincRNAs) have different functions.*

The manuscript has several grammatical mistakes, starting from the very first sentence in the Introduction section. Many paragraphs have loose statements and irrelevant information. E.g. See line 116 to 120. LincRNA and lncRNA are used interchangeably in many places.

Authors’ response: *We really appreciate the reviewer’s advice. We have revised the whole paper (including introduction) and deleted some irrelevant information.*

The methods section dealing with RNAfold need to be elaborated. Figures need to have proper units and labelling. e.g. MFE units.

Authors’ response: *It has been revised according to the reviewer’s suggestion.*

### Reviewer #3 (Second Round): Prof Manju Bansal, Indian Institute of Science, India

I had requested the author to explain the purpose of dividing the dataset into Overlapping and Unique. See earlier comment “Further, the dataset is divided into overlapping and unique. Stability study shows that overlapping lincRNAs are more stable than unique lincRNAs. The explanation for the observed difference is not clearly stated. I cannot understand why lincRNAs identified in two datasets (overlapping) should be more stable”. The author’s explanations are not satisfactory and have merely reiterated the aim of the work.

Authors’ response: *The lincRNAs are considered to play very important roles in gene regulation which shows dynamic properties in cellular processes. Therefore, identification of lincRNA stability is important to annotate its functions. In this paper, we classified the lincRNAs into overlapping lincRNAs and unique lincRNAs by comparing two RNA-Seq datasets of the same K562 cell line with venn figure (Figure*2*). In general, it is easy to understand that the overlapping lincRNAs are more stable than the unique lincRNAs, because the expressing probability of lincRNA should have high value if it could be observed in two separated experiments from ENCODE and our own detection in K562 cells. That it, lincRNAs expressed in both experiments statistically have higher expressing probability than lincRNAs only expressed in one single experiment. We have carried out RNA-Seq of K562 cell line, compared our RNA-Seq dataset with the corresponding ENCODE dataset, and found that a large proportion of protein-coding RNAs (86.7%) of our dataset appeared in ENCODE, but relative small proportion of lincRNAs (44.1%) of our dataset appeared in ENCODE (Figure*7*). We speculated this phenomenon arises due to the instability of lincRNAs during the cellular processes. We classified the overlapping part (overlapping lincRNAs) and the unique part (unique lincRNAs) from two datasets, and compared their stabilities. We have proved that a large proportion of overlapping lincRNAs were more stable than the unique lincRNAs through the analysis of differences in minimum free energy distribution and lincRNA half-lives.*

The authors postulate that the unique lincRNAs present in ENCODE or PH dataset should be functionally different. This implies that the RNAs that are identified and annotated as lincRNAs by both ENCODE and PH pipeline have similar function and this not true.

Authors’ response: *We suggested that overlapping lincRNAs (relatively stable linRNAs) and unique lincRNAs (relatively unstable lincRNAs) can be related to different functions, because they might be related to different cellular processes.*Further, minimum free energy of RNA is dependent on the GC content. A comment on the GC content of the overlapping and unique lincRNAs can explain the observed difference between the groups (Figure 3). Similar observation is warranted for half-life based stability analysis. If the GC content of the randomly selected lincRNAs in the two groups (overlapping (10 sequence) and unique (7 sequence)) is different then the selection of lincRNAs is biased by the sequence property. Moreover, mRNA expression is known to be affected by GC content. (G Kudla - ‎2006, PloS Biology.). Authors can check this phenomenon by binning the lincRNAs based on GC content and correlated with the level of expression.

Authors’ response: *In the previous studies, there is no correlation between lncRNA expression and its stability, although the signification correlation has been found for all RNAs (e.g. Clark et al. Genome Research, 2012, 22:885-898).*

PH dataset: full form of the abbreviation is given in the end (Methods section).

But the first mention is in ‘Introduction’ (line 124).

Authors’ response: *We have added the abbreviation of PH dataset directly in the introduction section where we first mentioned it.*

FPKM: the significance and full form of FPKM is not mention (Line 146)

Authors’ response: *We have added the full form of FPKM in revised version according to the reviewer’s suggestion.*

Line 82: ‘beacause’

Line 140-141: ‘were remained to carry out’

Line 147: ‘annotatied’

Authors’ response: *We have corrected the above spelling and grammar mistakes in the revised version according to the reviewer’s instruction.*

## Abbreviations

lincRNAs: Long intergenic non-coding RNAs; RNA-Seq: RNA Sequencing; lncRNAs: Long non-coding RNAs; ENCODE: The Encyclopedia of DNA Elements; TUCPs: The transcripts of uncertain coding potential; FPKM: Fragments Per Kilobase of exon model per Million mapped fragments; ORF: Open reading frame; NMD: Nonsense mediated decay; qPCR: Quantitative polymerase chain reaction; ActD: Actinomycin D; miRNA: microRNAs.

## Competing interests

The authors declare that they have no competing interests.

## Authors’ contributions

ZL conceived this study. LW and DZ designed the experimental plan, drafted and revised the manuscript. DZ carried out the field experiment. JT and YW participated in preparing, treating and collecting samples. LW analyzed and interpreted the sequence data. All authors read and approved the final manuscript.

## Supplementary Material

Additional file 1: Table S1 The bed file of the unique lincRNAs in PH. Click here for file

Additional file 2: Table S2 The bed file of the unique lincRNAs in ENCODE. Click here for file

Additional file 3: Table S3 The bed file of the overlapping lincRNAs of both datasets. Click here for file

Additional file 4: Table S4 The result for running Cuffcompare script to compare lincRNAs of both datasets. Click here for file

## References

[B1] Djebali S Davis CA Merkel A Dobin A Lassmann T Mortazavi A Tanzer A Lagarde J Lin W Schlesinger F Xue C Marinov GK Khatun J Williams BA Zaleski C Rozowsky J Roder M Kokocinski F Abdelhamid RF Alioto T Antoshechkin I Baer MT Bar NS Batut P Bell K Bell I Chakrabortty S Chen X Chrast J Curado J Landscape of transcription in human cells Nature 2012 489 101 108 10.1038/nature11233 22955620 PMC3684276

[B2] Yang L Froberg JE Lee JT Long noncoding RNAs: fresh perspectives into the RNA world Trends Biochem Sci 2014 39 35 43 10.1016/j.tibs.2013.10.002 24290031 PMC3904784

[B3] Ma L Bajic VB Zhang Z On the classification of long non-coding RNAs RNA Biol 2013 10 6 5 10.4161/rna.24604 PMC4111732 23696037

[B4] Cabili MN Trapnell C Goff L Koziol M Tazon-Vega B Regev A Rinn JL Integrative annotation of human large intergenic noncoding RNAs reveals global properties and specific subclasses Gene Dev 2011 25 1915 1927 10.1101/gad.17446611 21890647 PMC3185964

[B5] Ng JH Ng HH LincRNAs join the pluripotency alliance Nat Genet 2010 42 1035 1036 10.1038/ng1210-1035 21102618

[B6] Kumar V Westra HJ Karjalainen J Zhernakova DV Esko T Hrdlickova B Almeida R Zhernakova A Reinmaa E Vosa U Hofker MH Fehrmann RS Fu J Withoff S Metspalu A Franke L Wijmenga C Human disease-associated genetic variation impacts large intergenic non-coding RNA expression PLoS Genet 2013 9 e1003201 10.1371/journal.pgen.1003201 23341781 PMC3547830

[B7] Zhang X Lian Z Padden C Gerstein MB Rozowsky J Snyder M Gingeras TR Kapranov P Weissman SM Newburger PE A myelopoiesis-associated regulatory intergenic noncoding RNA transcript within the human HOXA cluster Blood 2009 113 2526 2534 10.1182/blood-2008-06-162164 19144990 PMC2656274

[B8] Huarte M Guttman M Feldser D Garber M Koziol MJ Kenzelmann-Broz D Khalil AM Zuk O Amit I Rabani M Attardi LD Regev A Lander ES Jacks T Rinn JL A large intergenic noncoding RNA induced by p53 mediates global gene repression in the p53 response Cell 2010 142 409 419 10.1016/j.cell.2010.06.040 20673990 PMC2956184

[B9] Hu WQ Yuan BB Flygare J Lodish HF Long noncoding RNA-mediated anti-apoptotic activity in murine erythroid terminal differentiation Gene Dev 2011 25 2573 2578 10.1101/gad.178780.111 22155924 PMC3248679

[B10] Khalil AM Guttman M Huarte M Garber M Raj A Rivea Morales D Thomas K Presser A Bernstein BE Van Oudenaarden A Regev A Lander ES Rinn JL Many human large intergenic noncoding RNAs associate with chromatin-modifying complexes and affect gene expression Proc Natl Acad Sci U S A 2009 106 11667 11672 10.1073/pnas.0904715106 19571010 PMC2704857

[B11] Ulitsky I Bartel DP lincRNAs: Genomics, Evolution, and Mechanisms Cell 2013 154 26 46 10.1016/j.cell.2013.06.020 23827673 PMC3924787

[B12] Orom UA Derrien T Beringer M Gumireddy K Gardini A Bussotti G Lai F Zytnicki M Notredame C Huang Q Guigo R Shiekhattar R Long noncoding RNAs with enhancer-like function in human cells Cell 2010 143 46 58 10.1016/j.cell.2010.09.001 20887892 PMC4108080

[B13] Bolognani F Perrone‒Bizzozero NI RNA–protein interactions and control of mRNA stability in neurons J Neurosci Res 2008 86 481 489 10.1002/jnr.21473 17853436

[B14] Rabani M Levin JZ Fan L Adiconis X Raychowdhury R Garber M Gnirke A Nusbaum C Hacohen N Friedman N Metabolic labeling of RNA uncovers principles of RNA production and degradation dynamics in mammalian cells Nat Biotechnol 2011 29 436 442 10.1038/nbt.1861 21516085 PMC3114636

[B15] Alonso CR A complex ‘mRNA degradation code’controls gene expression during animal development Trends Genet 2012 28 78 88 10.1016/j.tig.2011.10.005 22257633

[B16] Tani H Akimitsu N Genome-wide technology for determining RNA stability in mammalian cells: historical perspective and recent advantages based on modified nucleotide labeling RNA Biol 2012 9 1233 1238 10.4161/rna.22036 23034600 PMC3583853

[B17] Yildirim E Kirby JE Brown DE Mercier FE Sadreyev RI Scadden DT Lee JT Xist RNA is a potent suppressor of hematologic cancer in mice Cell 2013 152 727 742 10.1016/j.cell.2013.01.034 23415223 PMC3875356

[B18] Gabory A Jammes H Dandolo L The H19 locus: Role of an imprinted non‒coding RNA in growth and development Bioessays 2010 32 473 480 10.1002/bies.200900170 20486133

[B19] Tani H Mizutani R Salam KA Tano K Ijiri K Wakamatsu A Isogai T Suzuki Y Akimitsu N Genome-wide determination of RNA stability reveals hundreds of short-lived noncoding transcripts in mammals Genome Res 2012 22 947 956 10.1101/gr.130559.111 22369889 PMC3337439

[B20] Imamachi N Tani H Mizutani R Imamura K Irie T Suzuki Y Akimitsu N BRIC-seq: A genome-wide approach for determining RNA stability in mammalian cells Methods 2014 67 55 63 10.1016/j.ymeth.2013.07.014 23872059

[B21] Clark MB Johnston RL Inostroza-Ponta M Fox AH Fortini E Moscato P Dinger ME Mattick JS Genome-wide analysis of long noncoding RNA stability Genome Res 2012 22 885 898 10.1101/gr.131037.111 22406755 PMC3337434

[B22] Sharova LV Sharov AA Nedorezov T Piao Y Shaik N Ko MS Database for mRNA half-life of 19 977 genes obtained by DNA microarray analysis of pluripotent and differentiating mouse embryonic stem cells DNA Res 2009 16 45 58 10.1093/dnares/dsn030 19001483 PMC2644350

[B23] Yang E van Nimwegen E Zavolan M Rajewsky N Schroeder M Magnasco M Darnell JE Jr Decay rates of human mRNAs: correlation with functional characteristics and sequence attributes Genome Res 2003 13 1863 1872 12902380 10.1101/gr.1272403 PMC403777

[B24] Sutherland JA Turner RA Mannoni P McGann LE Turc J-M Differentiation of K562 leukemia cells along erythroid, macrophage, and megakaryocyte lineages J Immunother 1986 5 250 262 2425057

[B25] Guttman M Donaghey J Carey BW Garber M Grenier JK Munson G Young G Lucas AB Ach R Bruhn L Yang X Amit I Meissner A Regev A Rinn JL Root DE Lander ES lincRNAs act in the circuitry controlling pluripotency and differentiation Nature 2011 477 295 300 10.1038/nature10398 21874018 PMC3175327

[B26] Paralkar V Weiss M Long noncoding RNAs in biology and hematopoiesis Blood 2013 121 4842 4846 10.1182/blood-2013-03-456111 23645840 PMC3682336

[B27] Flicek P Amode MR Barrell D Beal K Billis K Brent S Carvalho-Silva D Clapham P Coates G Fitzgerald S Gil L Giron CG Gordon L Hourlier T Hunt S Johnson N Juettemann T Kahari AK Keenan S Kulesha E Martin FJ Maurel T McLaren WM Murphy DN Nag R Overduin B Pignatelli M Pritchard B Pritchard E Riat HS Ensembl 2014 Nucleic Acids Res 2013 42 D749 D755 24316576 10.1093/nar/gkt1196 PMC3964975

[B28] Meyer LR Zweig AS Hinrichs AS Karolchik D Kuhn RM Wong M Sloan CA Rosenbloom KR Roe G Rhead B Raney BJ Pohl A Malladi VS Li CH Lee BT Learned K Kirkup V Hsu F Heitner S Harte RA Haeussler M Guruvadoo L Goldman M Giardine BM Fujita PA Dreszer TR Diekhans M Cline MS Clawson H Barber GP The UCSC Genome Browser database: extensions and updates 2013 Nucleic Acids Res 2013 41 D64 D69 10.1093/nar/gks1048 23155063 PMC3531082

[B29] Harrow J Frankish A Gonzalez JM Tapanari E Diekhans M Kokocinski F Aken BL Barrell D Zadissa A Searle S Barnes I Bignell A Boychenko V Hunt T Kay M Mukherjee G Rajan J Despacio-Reyes G Saunders G Steward C Harte R Lin M Howald C Tanzer A Derrien T Chrast J Walters N Balasubramanian S Pei B Tress M GENCODE: the reference human genome annotation for The ENCODE Project Genome Res 2012 22 1760 1774 10.1101/gr.135350.111 22955987 PMC3431492

[B30] Pruitt KD Tatusova T Maglott DR NCBI reference sequences (RefSeq): a curated non-redundant sequence database of genomes, transcripts and proteins Nucleic Acids Res 2007 35 D61 D65 10.1093/nar/gkl842 17130148 PMC1716718

[B31] Sun K Chen X Jiang P Song X Wang H Sun H iSeeRNA: identification of long intergenic non-coding RNA transcripts from transcriptome sequencing data BMC Genomics 2013 14 S7 23445546 10.1186/1471-2164-14-S2-S7 PMC3582448

[B32] Wang L Park HJ Dasari S Wang S Kocher J-P Li W CPAT: Coding-Potential Assessment Tool using an alignment-free logistic regression model Nucleic Acids Res 2013 41 e74 10.1093/nar/gkt006 23335781 PMC3616698

[B33] Kong L Zhang Y Ye ZQ Liu XQ Zhao SQ Wei L Gao G CPC: assess the protein-coding potential of transcripts using sequence features and support vector machine Nucleic Acids Res 2007 35 W345 W349 10.1093/nar/gkm391 17631615 PMC1933232

[B34] Lin MF Jungreis I Kellis M PhyloCSF: a comparative genomics method to distinguish protein coding and non-coding regions Bioinformatics 2011 27 i275 i282 10.1093/bioinformatics/btr209 21685081 PMC3117341

[B35] Sun L Zhang Z Bailey TL Perkins AC Tallack MR Xu Z Liu H Prediction of novel long non-coding RNAs based on RNA-Seq data of mouse Klf1 knockout study BMC Bioinform 2012 13 331 10.1186/1471-2105-13-331 PMC3577497 23237380

[B36] Hangauer MJ Vaughn IW McManus MT Pervasive Transcription of the Human Genome Produces Thousands of Previously Unidentified Long Intergenic Noncoding RNAs PLoS Genet 2013 9 e1003569 10.1371/journal.pgen.1003569 23818866 PMC3688513

[B37] Managadze D Lobkovsky AE Wolf YI Shabalina SA Rogozin IB Koonin EV The vast, conserved mammalian lincRNome PLoS Comput Biol 2013 9 e1002917 10.1371/journal.pcbi.1002917 23468607 PMC3585383

[B38] Maenner S Blaud M Fouillen L Savoye A Marchand V Dubois A Sanglier-Cianferani S Van Dorsselaer A Clerc P Avner P Visvikis A Branlant C 2-D structure of the A region of Xist RNA and its implication for PRC2 association PLoS Biol 2010 8 e1000276 10.1371/journal.pbio.1000276 20052282 PMC2796953

[B39] Novikova IV Hennelly SP Sanbonmatsu KY Structural architecture of the human long non-coding RNA, steroid receptor RNA activator Nucleic Acids Res 2012 40 5034 5051 10.1093/nar/gks071 22362738 PMC3367176

[B40] Wilusz JE Jnbaptiste CK Lu LY Kuhn CD Joshua-Tor L Sharp PA A triple helix stabilizes the 3' ends of long noncoding RNAs that lack poly(A) tails Gene Dev 2012 26 2392 2407 10.1101/gad.204438.112 23073843 PMC3489998

[B41] Mercer TR Mattick JS Structure and function of long noncoding RNAs in epigenetic regulation Nat Struct Mol Biol 2013 20 300 307 10.1038/nsmb.2480 23463315

[B42] Clote P Ferre F Kranakis E Krizanc D Structural RNA has lower folding energy than random RNA of the same dinucleotide frequency RNA 2005 11 578 591 10.1261/rna.7220505 15840812 PMC1370746

[B43] Dinger ME Amaral PP Mercer TR Mattick JS Pervasive transcription of the eukaryotic genome: functional indices and conceptual implications Brief Funct Genomic Proteomic 2009 8 407 423 10.1093/bfgp/elp038 19770204

[B44] Dinger ME Amaral PP Mercer TR Pang KC Bruce SJ Gardiner BB Askarian-Amiri ME Ru K Solda G Simons C Sunkin SM Crowe ML Grimmond SM Perkins AC Mattick JS Long noncoding RNAs in mouse embryonic stem cell pluripotency and differentiation Genome Res 2008 18 1433 1445 10.1101/gr.078378.108 18562676 PMC2527704

[B45] Schwanhausser B Busse D Li N Dittmar G Schuchhardt J Wolf J Chen W Selbach M Global quantification of mammalian gene expression control Nature 2011 473 337 342 10.1038/nature10098 21593866

[B46] Froberg JE Yang L Lee JT Guided by RNAs: X-Inactivation as a Model for lncRNA Function J Mol Biol 2013 425 3698 3706 10.1016/j.jmb.2013.06.031 23816838 PMC3771680

[B47] Wilusz JE Freier SM Spector DL 3' end processing of a long nuclear-retained noncoding RNA yields a tRNA-like cytoplasmic RNA Cell 2008 135 919 932 10.1016/j.cell.2008.10.012 19041754 PMC2722846

[B48] Amaral PP Clark MB Gascoigne DK Dinger ME Mattick JS lncRNAdb: a reference database for long noncoding RNAs Nucleic Acids Res 2011 39 D146 D151 10.1093/nar/gkq1138 21112873 PMC3013714

[B49] Friedel CC Dölken L Ruzsics Z Koszinowski UH Zimmer R Conserved principles of mammalian transcriptional regulation revealed by RNA half-life Nucleic Acids Res 2009 37 e115 115 10.1093/nar/gkp542 19561200 PMC2761256

[B50] Gupta RA Shah N Wang KC Kim J Horlings HM Wong DJ Tsai MC Hung T Argani P Rinn JL Wang Y Brzoska P Kong B Li R West RB van de Vijver MJ Sukumar S Chang HY Long non-coding RNA HOTAIR reprograms chromatin state to promote cancer metastasis Nature 2010 464 1071 1076 10.1038/nature08975 20393566 PMC3049919

[B51] Kino T Hurt DE Ichijo T Nader N Chrousos GP Noncoding RNA gas5 is a growth arrest- and starvation-associated repressor of the glucocorticoid receptor Sci Signal 2010 3 ra8 20124551 10.1126/scisignal.2000568 PMC2819218

[B52] Keniry A Oxley D Monnier P Kyba M Dandolo L Smits G Reik W The H19 lincRNA is a developmental reservoir of miR-675 that suppresses growth and Igf1r Nat Cell Biol 2012 14 659 665 10.1038/ncb2521 22684254 PMC3389517

[B53] Bolger AM Lohse M Usadel B Trimmomatic: A flexible trimmer for Illumina Sequence Data Bioinformatics 2014 doi: 10.1093/bioinformatics/btu170 10.1093/bioinformatics/btu170 PMC4103590 24695404

[B54] Kim D Pertea G Trapnell C Pimentel H Kelley R Salzberg SL TopHat2: accurate alignment of transcriptomes in the presence of insertions, deletions and gene fusions Genome Biol 2013 14 R36 10.1186/gb-2013-14-4-r36 23618408 PMC4053844

[B55] Trapnell C Hendrickson DG Sauvageau M Goff L Rinn JL Pachter L Differential analysis of gene regulation at transcript resolution with RNA-seq Nat Biotechnol 2013 31 46 53 23222703 10.1038/nbt.2450 PMC3869392

[B56] Wang L Wang S Li W RSeQC: quality control of RNA-seq experiments Bioinformatics 2012 28 2184 2185 10.1093/bioinformatics/bts356 22743226

[B57] Zuker M Stiegler P Optimal computer folding of large RNA sequences using thermodynamics and auxiliary information Nucleic Acids Res 1981 9 133 148 10.1093/nar/9.1.133 6163133 PMC326673

[B58] Hofacker IL Vienna RNA secondary structure server Nucleic Acids Res 2003 31 3429 3431 10.1093/nar/gkg599 12824340 PMC169005

